# Initial characterization, dosimetric benchmark and performance validation of Dynamic Wave Arc

**DOI:** 10.1186/s13014-016-0633-7

**Published:** 2016-04-29

**Authors:** Manuela Burghelea, Dirk Verellen, Kenneth Poels, Cecilia Hung, Mitsuhiro Nakamura, Jennifer Dhont, Thierry Gevaert, Robbe Van den Begin, Christine Collen, Yukinori Matsuo, Takahiro Kishi, Viorica Simon, Masahiro Hiraoka, Mark de Ridder

**Affiliations:** Department of Radiotherapy, Universitair Ziekenhuis Brussel, Vrije Universiteit Brussel, Laarbeeklaan 101, B-1090 Brussels, Belgium; Department of Radiation Oncology, University Hospitals Leuven, Leuven, Belgium; Department of Radiation Oncology and Image-applied Therapy, Kyoto University Graduate School of Medicine, Kyoto, Japan; R&D Radiosurgery, BrainLAB AG, Munich, Germany; Babes Bolyai University, Faculty of Physics, Cluj-Napoca, Romania

**Keywords:** Dynamic Wave Arc, Noncoplanar delivery, 2D diode dosimeter

## Abstract

**Background:**

Dynamic Wave Arc (DWA) is a clinical approach designed to maximize the versatility of Vero SBRT system by synchronizing the gantry-ring noncoplanar movement with D-MLC optimization. The purpose of this study was to verify the delivery accuracy of DWA approach and to evaluate the potential dosimetric benefits.

**Methods:**

DWA is an extended form of VMAT with a continuous varying ring position. The main difference in the optimization modules of VMAT and DWA is during the angular spacing, where the DWA algorithm does not consider the gantry spacing, but only the Euclidian norm of the ring and gantry angle. A preclinical version of RayStation v4.6 (RaySearch Laboratories, Sweden) was used to create patient specific wave arc trajectories for 31 patients with various anatomical tumor regions (prostate, oligometatstatic cases, centrally-located non-small cell lung cancer (NSCLC) and locally advanced pancreatic cancer-LAPC). DWA was benchmarked against the current clinical approaches and coplanar VMAT. Each plan was evaluated with regards to dose distribution, modulation complexity (MCS), monitor units and treatment time efficiency. The delivery accuracy was evaluated using a 2D diode array that takes in consideration the multi-dimensionality of DWA during dose reconstruction.

**Results:**

In centrally-located NSCLC cases, DWA improved the low dose spillage with 20 %, while the target coverage was increased with 17 % compared to 3D CRT. The structures that significantly benefited from using DWA were proximal bronchus and esophagus, with the maximal dose being reduced by 17 % and 24 %, respectively. For prostate and LAPC, neither technique seemed clearly superior to the other; however, DWA reduced with more than 65 % of the delivery time over IMRT. A steeper dose gradient outside the target was observed for all treatment sites (*p* < 0.01) with DWA. Except the oligometastatic cases, where the DWA-MCSs indicate a higher modulation, both DWA and VMAT modalities provide plans of similar complexity. The average ɣ (3 % /3 mm) passing rate for DWA plans was 99.2 ± 1 % (range from 96.8 to 100 %).

**Conclusions:**

DWA proven to be a fully functional treatment technique, allowing additional flexibility in dose shaping, while preserving dosimetrically robust delivery and treatment times comparable with coplanar VMAT.

**Electronic supplementary material:**

The online version of this article (doi:10.1186/s13014-016-0633-7) contains supplementary material, which is available to authorized users.

## Background

In the past few years, there has been a widespread adoption of rotational beam-delivery techniques into the clinic. With more degrees of freedom during treatment, advanced arc deliveries provide a larger flexibility in shaping dose distributions while obtaining more time-efficient deliveries than with stationary beams. Currently, volumetric modulated arc therapy (VMAT) is one of the most advanced delivery techniques, simultaneously integrating multi-leaf collimator (MLC) field shape modulation with gantry speed and dose rate variation. Dose delivery by VMAT may further be optimized by increasing the degrees-of-freedom. More specifically, technological evolutions have brought the use of noncoplanar arc delivery within reach of clinical application.

For noncoplanar VMAT by dynamic couch rotation, several groups have already reported dosimetric advantages for different treatment sites, hereby the couch and gantry following either a user-defined trajectory [1, 2] or a directly optimized trajectory [3–6]. However, to the authors’ knowledge, none of the stated delivery approaches are commercially available or implemented in a conventional clinical workflow.

Dynamic Wave Arc (DWA) is a clinical arc approach designed to maximize the noncoplanar versatility of Vero SBRT system by combining the gantry-ring synchronized rotation with D-MLC optimization. The speed of the gantry and ring can be accurately modulated due to the rigid gear transmission design, which allows the system to preserve its rigidity even during complex gantry-ring movements. The theoretical concept of DWA was presented by Mizowaki et al. [7] under the term “3D unicursal irradiation” and the technique was subject to other more technically orientated reports regarding geometrical and dosimetric accuracy [8, 9].

This paper describes the implementation of DWA following a standard clinical workflow by integrating a dedicated commercially-available treatment planning system (TPS) on the Vero SBRT treatment platform. The optimization algorithm for DWA planning was validated and characterized by comparing it with the standard coplanar VMAT optimizer. The first results of applying DWA on different treatment sites are presented. Subsequently the DWA/VMAT delivery accuracy was evaluated using a diode array which takes the noncoplanar gantry-ring trajectory in consideration during dose reconstruction.

## Methods

### A. Patient selection

Thirty-one patients with various anatomical tumor regions were selected from the Vero database. Based on its high incidence for radiation therapy, prostate cancer and SBRT for oligometastases pathologies were selected for this study. Additionally, some more challenging cases regarding the avoidance of organs-at-risk (OAR), i.e. centrally-located non-small cell lung cancer (NSCLC) and locally-advanced pancreatic cancer (LAPC) were added considering that a maximum OAR sparing can potentially reduce treatment-toxicity. Table 1 summarizes the treatment indications and fractionation, along with the main OAR dose limitations. For the prostate, the clinical target includes the prostate gland with the base of seminal vesicles. The PTV was created adding a uniform 6 mm expansion, while the rectum was the main critical OAR. The oligometastatic patients are part of a prospective clinical trial [10], and include multiple (≤3) lung and liver lesions. The planning constraints of SBRT for centrally-located NSCLC were adopted from the RTOG 0813 guidelines. The pancreatic cases were outlined using the ITV approach [11, 12]. The dose-limiting OARs (duodenum, stomach) were contoured with a planning risk margin of 3 mm to accomplish the gastro-intestinal dose constraints.

**Table 1:** Overview of the patient data related to treatment indication and fractionation. The physics dose limitations for the most common OARs are included together with the delivery technique clinically used in our center

Site	Nr. of patients		Fractionation	Minimum % of PD covering PTV	PTV Volume (ccm)	OAR	Dose constrains	Clinical delivery technique
Prostate	3		78Gy(2Gy/fx)	>90 %	171.8 [129.8-222.8]	Rectum	D_2%_ < 74.1 Gy V_70.2Gy_ < 25 % V_62.4Gy_ < 50 %	5 IMRT beams
Oligo-metastatic cases	9	5 x1lesion 2x2lesions 2x3lesions	50Gy^a^(5Gy/fx)	100 %	28.0 [12.2-57.5]	Lung Liver	V_20Gy_ < 25 % V_30Gy_ < 30 % D_mean_ < 22 Gy	8-10 CRT beams per lesion
Centrally-located NSCLC	9	6 lesions 3 lesions	48Gy^b^(12Gy/fx) 60Gy(7.5Gy/fx)	>95 % >95 %	76.2 [17.2-209.7]	Esophagus Spinal cord Heart Proximal- bronchus Lung	D_2%_ < 30 Gy D_2%_ < 26 Gy D_2%_ < 34 Gy D_2%_ < 34 Gy V_20Gy_ < (8-10)%	8-10 CRT beams
LAPC	10		48Gy(3.2Gy/fx)	>95 %	188.5 [140.6-257.2]	Duodenum	V_39Gy_ < 1.0 cc V_36Gy_ < 10 cc	6 IMRT beams
						Stomach Kidney Liver	V_39Gy_ < 1.0 cc V_36Gy_ < 10 cc V_20Gy_ < 30 % D_mean_ < 30 Gy	

^a^ *at 80%isodose*

^*b*^ *variable isodose prescriptions*

*Abbreviations: NSCLC*, centrally-located non-small cell lung cancer; *LAPC*, locally-advanced pancreatic cancer; *PD*, prescription dose; *D*_*2%*_ near-maximum dose i.e. maximum dose to 2 % of the volume; *V*_*xx%*_, percentage of the volume receiving ≥ xx Gy; *V*_*xxGy*_ volume of the structure receiving ≥ xx Gy; *D*_*mean*_, mean dose

### B. DWA characterization

A preclinical version of RayStation v4.6 (RaySearch Laboratories, Sweden) was used to create patient-specific DWA trajectories on the Vero SBRT system in order to develop site-specific class solutions for the final product. A wave trajectory is defined by 5 to 8 manipulation points (MPs) that are gantry-ring angle positions where the direction of the ring rotation can change. The MPs were selected to minimize overlap between PTV and organs at risk through beam's eye view (BEV) inspection by a human planner, while remaining within the ring-table collision limitations provided by the manufacturer.

According to the TPS vendor’s specifications, the dominant angle is either the ring or gantry angle, depending on which has the largest angle span, and is determined per sub-arc (defined by two consecutive MPs). The dominant angle should be a multiple of 4° and the minimum angular distance per sub-arc should be 24°. An additional constraint of 1 degree ring rotation per gantry degree was enforced in the optimization algorithm, to avoid creating too “steep” sub-arcs in terms of ring rotation with respect to gantry rotation.

DWA is an extended form of VMAT with a continuous variable ring position. The user sets up the dose-volume objective functions in the same way as VMAT, however the optimization starts with beam generation at 24° increments along the predefined wave trajectory. A fluence map optimization follows and, at each initial angle, 2-4 segments are generated and filtered so that there are only two control points (CP) per beam angle. The CPs are linearly interpolated from the adjacent CPs and equally distributed between them. After the MLC segments are created, a direct machine parameter optimization (DMPO) (13) algorithm is applied considering the following DWA specific machine constraints: gantry speed (0.1-6°/s), ring speed (0.1-2.5°/s), dose rate (150-400 MU/min), and MLC leaf speed (0.1–4 cm/s). The algorithm incorporates the dose rate into the optimization process by selecting its value from a discrete look-up table. While the ring speed is modulated per sub-arc, the optimizer assumes constant gantry speed and constant dose rate throughout the arc to achieve constant MU per CP. The dose calculation engine is based on the collapsed cone convolution superposition algorithm.

These machine constraints are similar for coplanar VMAT on the Vero system, i.e. constant dose rate and constant gantry speed (VMAT_CDR_) [13, 14]. The main difference in the optimization modules of VMAT_CDR_ and DWA is the angular spacing of control points, where the DWA algorithm does not consider the gantry spacing, but the Euclidian norm of the ring and gantry.

### C. Treatment scenarios and dosimetric benchmark

**Fig. 1:**
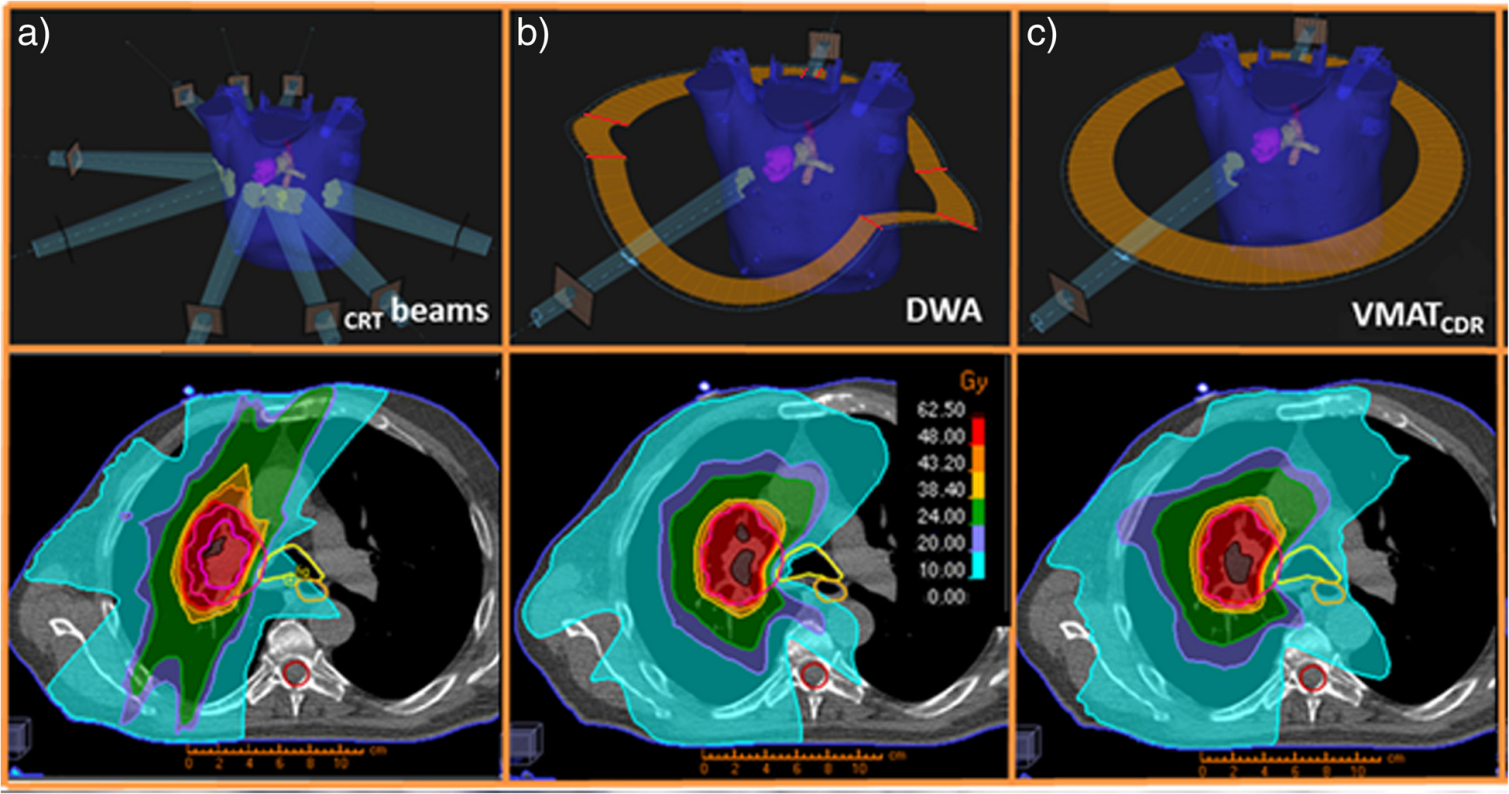
Treatment delivery scenarios investigated along with the dose distribution in the axial plane. **a**) coplanar/ noncoplanar static beams **b**) DWA noncoplanar trajectory defined by 7 MPs highlighted in red **c**) VMAT_CDR_ denotes a coplanar arc solution with MLC filed shape modulation, but constant gantry speed and constant dose rate

Figure 1 gives an overview of the treatment scenarios investigated, along with a comprehensive description for each one. The clinical delivery techniques presented in Table 1 were considered as reference and were compared with DWA and VMAT_CDR_.

The planning objectives for target volumes and OARs were individualized for each patient, but kept identical between VMAT_CDR_ and DWA. For multiple lesions, one arc per lesion was used and all targets were simultaneously optimized. Due to the PTV-OAR overlap and complex geometry in the LAPC cases, two independent DWA trajectories were customized mostly to take advantage of the gantry-ring collision-free space. For prostate, single-arc treatment was employed for both DWA and VMAT_CDR_.

Each plan was evaluated with regard to the target coverage, dose to OAR, monitor units (MU) and treatment delivery time. The PTV coverage was defined by the ratio between target volume within the prescription isodose and PTV volume. The low dose spillage describes the ratio between the volume receiving 50 % of the prescription dose and the PTV volume. The planning techniques were compared using a paired two-tailed Student’s *t*-test, with *p* < 0.05 considered as statistically significant.

### D. DWA performance validation

The DWA/ VMAT_CDR_ dosimetric tests were performed on a Vero research machine (BrainLAB, Germany) featuring a software and hardware upgrade to support DWA and VMAT deliveries. Essentially, the machine’s capability to perform a faster dynamic MLC leaf movement (i.e. 4 cm/s at isocenter level) was activated. The R&V system is able to recognize DWA plans, while the controller allows gantry-ring synchronous rotations.

The quality of the generated DWA plans was assessed using the Delta^4^ diode array phantom (ScandiDos, Sweden) [15–17] and a preclinical software version which supports automatically import of gantry-ring angular positions per CP from the DICOM RT plan. During measurement the dose pulses are sorted into these CPs according to the planned number of MU per CP. The corresponding angular correction factors are being applied for each CP during dose reconstruction and the agreement score with DWA dose from the TPS was derived by calculating the minimum 3D gamma index for each diode point in the orthogonal 2D planes. For a specific diode in the 2D planes, the software includes the full planned 3D dose in the whole phantom volume to find the minimum gamma index. A global 3 % dose difference and 3 mm distance-to-agreement criteria was used, with a lower threshold level of 20 % of the maximum expected dose.

The DWA/VMAT_CDR_ complexity was quantified by using the modulation complexity score (MCS) proposed by Masi et al. [18, 19]. The MCS computation takes in consideration the product of mean values between adjacent CPs of leaf sequence variability and aperture area variability, weighted by the relative MU delivered between two consecutive CPs and then summed over all CPs in the arc. Its value ranges between 0 and 1, where 1 means no complexity.

In order to clarify possible discrepancies between planning and physical machine performance, the TPS estimations were compared with the actual DWA LogFiles recorder by the machine’s controller during delivery at an increased sampling rate of 100Hz (~10 ms). The most relevant information in the DWA LogFile is the gantry-ring speed profiles and the dose rate profile. It is worth mentioning that in the TPS, the ring speed variation per sub-arc is integrated, while the gantry speed and dose rate are assumed constant over the entire wave. However, the controller recalculates and adjusts these three parameters at each MP in order to have the fastest delivery [20].

## Results

**Fig. 2:**
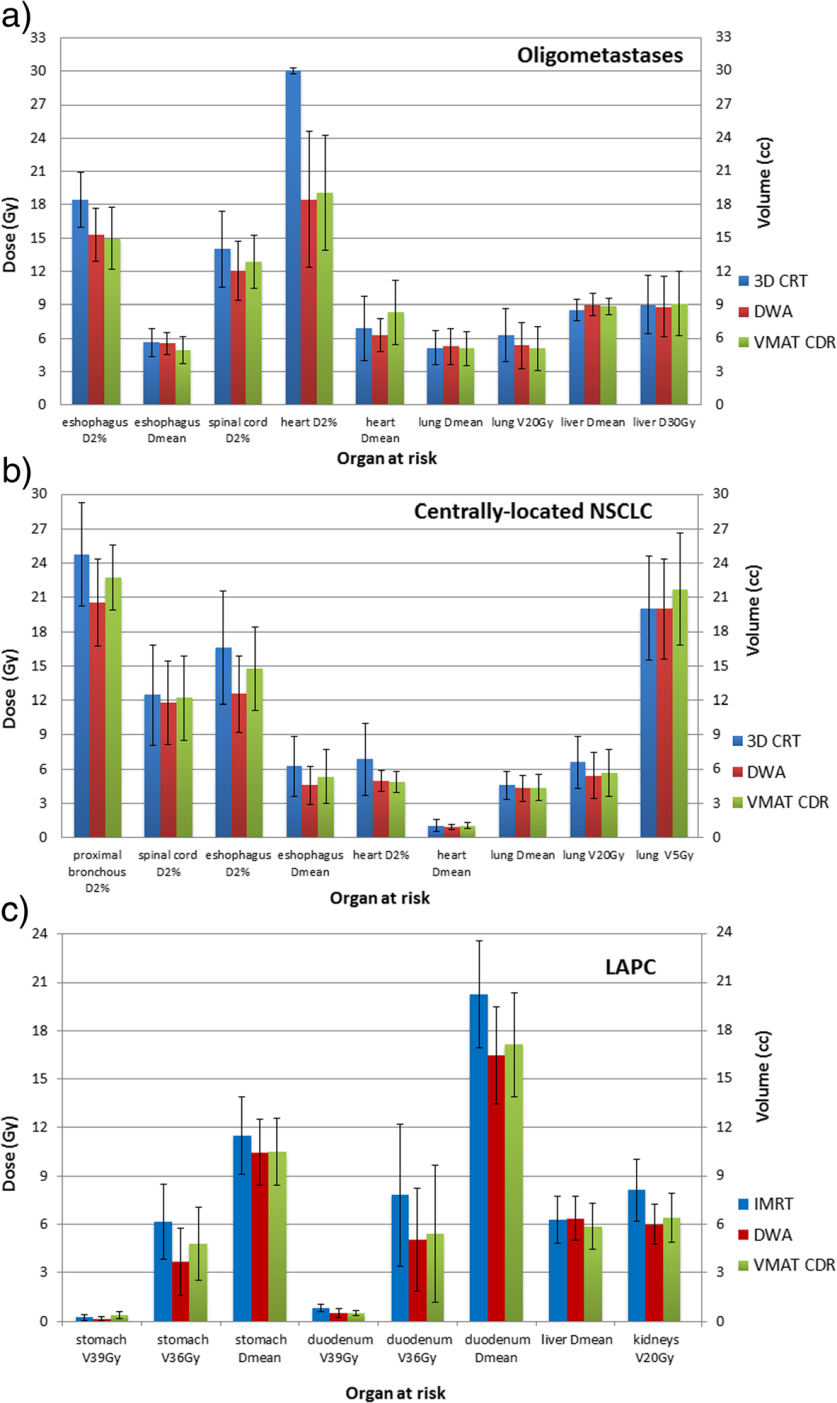
Dose statistics for the most common OARs in **a**) oligometastases, **b**) centrally-located NSCLC and **c**) LAPC

The dose statistics per indication are summarized in Table 2. Figure 2 shows the OARs diagrams for the different delivery approaches. The prostate cases presented comparable plan quality with no significant difference in PTV coverage. Similar values were found for the high doses to the rectum. Rectum V_70.2Gy_ was 9.7 %, 8.3 %, and 8.1 % for IMRT, DWA and VMAT_CDR_ respectively, while rectum V_62.4Gy_ was 22.0 %, 19.8 %, and 19.4 %. The arc-based techniques are able to deliver the dose faster, shortening the treatment time with more than 70 %.

**Table 2:** Summary of the results of the target volume dose distribution and delivery parameters for all investigated scenarios

Site		3D CRT/IMRT		DWA		VMAT_CDR_
		Mean ± SD	*p*-value	Mean ± SD	*p*-value	Mean ± SD
Prostate n = 3	Coverage PTV D_90%_	0.99 ± 0.01	*p* = 0.95	0.98 ± 0.03	*p* = 0.90	0.98 ± 0.03
	Low dose spillage	3.99 ± 0.21	*p* = 0.66	3.81 ± 0.22	*p* = 0.49	4.08 ± 0.58
	MU	531 ± 45	*p* = 0.24	495 ± 2	*p* = 0.36	463 ± 54
	Estimated time (min)	-	-	3.09 ± 0.02	*p* < 0.01	1.51 ± 0.13
	Actual time (min)	5.55 ± 0.45	*p* < 0.01	1.59 ± 0.01	*p* < 0.01	1.23 ± 0.11
Oligo- metastatic cases n = 15	Coverage PTV D_80%_	0.96 ± 0.1	*p* = 0.27	0.98 ± 0.05	*p* = 0.35	0.97 ± 0.07
	Low dose spillage	5.98 ± 2.33	*p* = 0.01	4.87 ± 1.23	*p* = 0.25	5.03 ± 1.03
	MU	975 ± 211	*p* < 0.01	1370 ± 346	*p* = 0.29	1320 ± 309
	Estimated time (min)	-	-	3.80 ± 1.41	*p* = 0.16	3.51 ± 0.80
	Actual time (min)	5.47 ± 1.04	*p* < 0.01	3.44 ± 0.88	*p* = 0.94	3.46 ± 0.88
Centrally- located NSCLC n = 9	Coverage PTV D_95%_	0.84 ± 0.20	*p* = 0.21	0.91 ± 0.06	*p* = 0.26	0.90 ± 0.09
	Low dose spillage	5.39 ± 1.24	*p* = 0.01	4.43 ± 1.06	*p* = 0.11	4.58 ± 1.02
	MU	1885 ± 477	*p* < 0.01	3349 ± 896	*p* = 0.29	3238 ± 809
	Estimated time (min)	-	-	8.36 ± 1.83	*p* = 0.61	8.36 ± 1.98
	Actual time (min)	7.08 ± 1.09	*p* = 0.18	8.45 ± 2.32	*p* = 0.90	8.59 ± 2.29
LAPC n = 10	Coverage PTV D_98%_	0.71 ± 0.23	*p* = 0.59	0.76 ± 0.13	*p* = 0.94	0.76 ± 0.15
	Coverage GTV D_95%_	0.81 ± 0.17	*p* = 0.53	0.85 ± 0.15	*p* = 0.10	0.85 ± 0.18
	Low dose spillage	3.70 ± 0.28	*p* < 0.01	3.25 ± 0.22	*p* = 0.90	3.34 ± 0.22
	MU	1091 ± 100	*p* < 0.01	644 ± 65	*p* = 0.10	704 ± 87
	Estimated time (min)	-	-	3.10 ± 0.15	*p* = 0.34	3.16 ± 0.20
	Actual time (min)	6.83 ± 0.41	*p* < 0.01	2.42 ± 0.01	*p* = 0.63	2.51 ± 0.28

Note: The data is presented as mean values ± SD. The statistical difference between 3D CRT vs. DWA and DWA vs. VMATCDR are presented in the first and second p-value columns, respectively Estimated time = TPS approximation based on the dose rate assigned during optimization; Actual time = measured beam-on delivery time.

For the oligometastases, all modalities provided comparable plan qualities, with no significant difference in PTV coverage or OAR sparing, but with an improved low dose spillage for DWA (*p* = 0.01) and comparable to VMAT_CDR_ (*p* > 0.05). Mean lung doses and V_20Gy_ presented minimal differences, same for mean liver doses and V_30Gy_ for the liver cases (Fig. 2a). The number of MU per lesion had an 41 % increase in DWA and VMAT_CDR_ cases versus 3D CRT; however the delivery time per lesion was reduced with 37 %.

Compared with 3D CRT, rotational techniques increased target coverage for centrally-located NSCLC, while keeping the risk structures below dose limits, at the cost of an increase in MUs of 75 % and delivery time of 20 % (Table 2). The structures significantly benefitting from DWA are proximal bronchus (*p* = 0.04) and esophagus (*p* = 0.05), while other OARs present comparable values (Fig. 2b). Compared with VMAT_CDR_, DWA reduced D_2%_ with a mean of 2Gy for proximal bronchus and esophagus. The clinical consideration of decreased efficacy versus possible benefit is of course a matter of debate. TPS estimated times were close to actual delivery times for both DWA and VMAT_CDR_.

In the LAPC cases, DWA achieved similar PTV and GTV coverage, with a significantly improved low dose distribution (*p* < 0.01) compared to IMRT. The number of MU was significantly lower for DWA versus IMRT, and comparable with VMAT_CDR_ (Table 2). The delivery time was reduced by 65 % and 63 % with DWA and VMAT_CDR_, respectively. The TPS time estimation was overestimated with approx. 21 % for both techniques. The duodenum V_36Gy_ volume is substantially reduced with DWA compared with IMRT (*p* = 0.01), but similar compared to VMAT_CDR_ (*p* = 0.39). No significant difference was observed for the stomach doses between the three approaches. The volume of both kidneys exceeding 20Gy was decreased by 26 % over IMRT using DWA (Fig. 2c).

The MCSs and gamma passing rates for DWA and VMAT_CDR_ are presented in Table 3. Overall investigated indications, the oligometastatic plans showed the highest complexity. The DWA-MCSs were significantly lower compared with VMAT_CDR_ –MCSs (*p* = 0.03). No meaningful difference was observed between MCSc of DWA and VMAT_CDR_ for centrally-located NSCLC (*p* = 0.34), prostate (*p* = 0.42) or LAPC (*p* = 0.29), respectively. The DWA plans presented a good agreement between measured and calculated dose, with an average ɣ(3 %/3 mm) passing rate of 99.2 ± 1 % (range from 96.8 % to 100 %). The VMAT_CDR_ average γ (3 %/3 mm) passing rate was 98.4 ± 1.4 % (range from 96.4 % to 100 %).

**Table 3:** Site summary for modulation complexity scores and gamma passing rates. The data is presented as mean values ± SD

		DWA	VMAT_CDR_	
		mean ± SD	mean ± SD	*p*-value
Prostate	MCS	0.46 ± 0.02	0.49 ± 0.06	*p* = 0.42
	Ɣ-index (%)	98.17 ± 0.86	98.4 ± 1.71	*p* = 0.97
Oligometastatic cases	MCS	0.28 ± 0.05	0.32 ± 0.05	*p* = 0.03
	Ɣ-index (%)	98.72 ± 1.13	98.42 ± 1.42	*p* = 0.08
Centrally- located NSCLC	MCS	0.33 ± 0.07	0.35 ± 0.09	*p* = 0.34
	Ɣ-index (%)	99.2 ± 0.91	98.54 ± 1.83	*p* = 0.96
LAPC	MCS	0.46 ± 0.04	0.44 ± 0.04	*p* = 0.29
	Ɣ-index (%)	98.1 ± 0.74	98.20 ± 0.70	*p* = 0.80

*Abbreviations: MCS*, modulation complexity scores; Ɣ (3 %/3 mm), index gamma passing rate representing the agreement score between calculated and measured dose distributions; *p*-value, statistical output of the student *t*-test

**Fig. 3:**
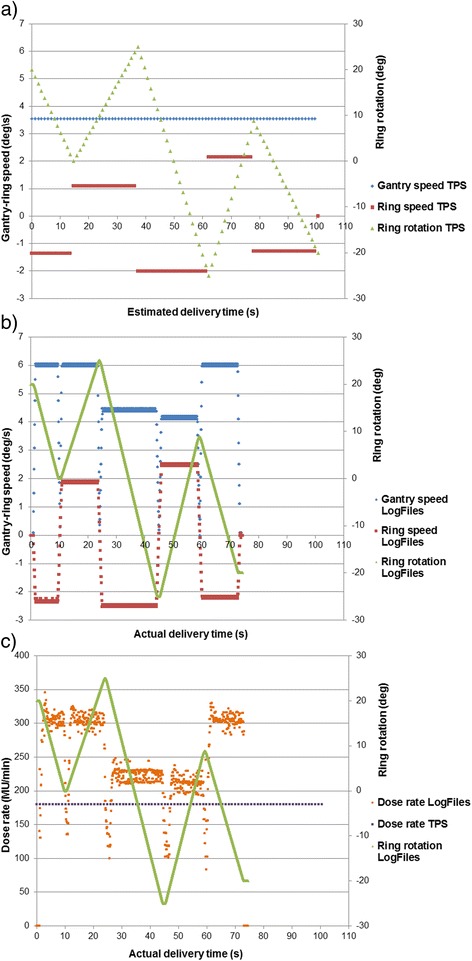
Angular gantry-ring velocity profile and dose rate profile for a pancreatic DWA plan (with 301MU). **a**) gantry-ring angular velocity in function of delivery time TPS estimated per CP **b**) gantry-ring angular velocity in function of delivery time acquired by the machine’s controller **c**) dose rate profile comparing TPS estimated and actual dose rate variation. The ring angular rotation was used as a common base in each figure to indicate the directional change during DWA delivery

Figure 3 presents the gantry-ring angular velocity estimated by the TPS (Fig. 3a) for a pancreatic case, benchmarked with the actual values recorded by the LogFiles in function of time (Fig. 3b). The mean gantry-ring speed values from the LogFiles were 4.78°/s and 2.99°/s, while the TPS estimated mean values were 3.55°/s and 1.55°/s, respectively. Figure 3c shows the dose rate discrepancy between TPS approximation (180MU/min) and actual used value (≈243 MU/min), difference that contributes to a delivery time overestimation of 26 %. The dose rate modulation behavior in synchrony with the mechanical stopping motion of the system, quantified to 0.1 s can also be observed in Fig. 3c.

## Discussion

Several investigators have reported that noncoplanar beam/arc arrangements improve dosimetric quality; however its clinical usability is considered rather controversial. Recently, Wild et al. [21] compared nine different treatment scenarios based on plan quality and delivery efficiency, and concluded that noncoplanar Step&Shoot IMRT beams and VMAT applying optimized, arbitrary, noncoplanar trajectories are valuable clinical approaches.

Clinical implementation of DWA is a viable option due to the following factors: added variable angles of orientation; no customized workflow required; delivery time comparable with coplanar VMAT_CDR_. The objections against couch rotation during VMAT delivery with an L-shape LINAC are mostly focused on possible patient movement during beam on, which is difficult to predict or adapt for. Re-verification of the patient setup after or during rotation of the couch is therefore currently required in clinical routine [22, 23]. The Wave Arc planning module allows one to design individualized patient treatments, while the Vero’s hardware controller ensures a continuous reliable delivery [8, 9]. No additional patient immobilization is required other than that already employed in clinical routine, since the O-ring design allows two-dimensional movements without any couch maneuvering. There are no patient-specific anti-collision rules enforced by the software, however this can be compensated by manual verification performed using the control pendant inside the treatment room to run wave trajectories without beam-on.

In comparison with 3D CRT, DWA presented a steeper dose fall-off and improved target coverage. In the centrally-located NSCLC cases, the mean reduction in normal tissue exposed to ≥50 % of the prescription dose was 20 %, while the target coverage was increased with 17 %. The direct aperture optimization is able to better shape the dose around targets, which are in close proximity or overlapping with one or more OARs, whereas the non-coplanarity maintains low doses to the lung. For prostate and LAPC, neither technique seemed clearly superior to the other. However, considering that Vero cannot perform a collimator rotation, DWA would be recommended in practice while treating these indications. The different ring rotations during DWA would “act as a collimation variation”, increasing the conformal shaping capabilities for irregular-shaped target volumes and at the same time minimizing the cumulative effects of the tongue-and-groove and interleaf transmission [24, 25]. The course of treatment was considerably shortened with DWA for all indications investigated, except for centrally-located NSCLC SBRT where it was slightly increased due to the higher MUs per fraction and the MLC sequence complexity.

The estimated treatment time was frequently longer than the actual time due to the conservative DWA module, that is optimized based on Vero machine’s specifications with constant dose rate and constant gantry speed. The delivery time presented in RayStation TPS for the Vero machine is not to be seen as the estimated delivery time, rather as a feasible delivery time. The gantry speed and ring speed displayed in the user interface are presented to give information on how the time dependence is modelled in the TPS. The optimizer creates a plan that is possible to deliver using the selected delivery time i.e. combination of gantry speed and dose rate. The controller of the Vero machine might (and will in most cases) select another gantry speed to optimize the delivery performance, independent of the TPS (which provides a deliverable plan with feasible MUs per CPs). A descriptive representation of the controller’s complex adjustments during DWA is presented in Fig. 3, where the dose rate and gantry-ring speed ratio readjustment per sub-arc can be observed. At each MP, the dose rate was reduced to ~100 MU/min, introducing an overall decrease in the cumulative MUs of 0.23MU with negligible effects on the actual dose. The Vero’s control system always aims for the fastest delivery, as such the highest dose rate is selected by default, which is decreased when ring speed is a limiting factor [20].

As DWA and VMAT_CDR_ employ nearly identical optimization modules, with the planning objectives being kept alike, the modest dosimetric improvements can be attributed to the noncoplanar geometry. It is worth mentioning that, from the total number of CPs per DWA, only approx. 30 % have a ring rotation larger than 15°. Wild et al. [21] results indicated that 9-beam ensemble were clearly superior compared to five, while 13-beam ensemble showed only small plan quality improvements. Dong et al.[26] showed that more than 10 couch kicks are needed to enlarge noncoplanar solution space and to have notable dose decrease.

DWA and VMAT_CDR_ modalities provide plans of similar complexity, MLC leaf velocity being the leading parameter during optimization. The DWA-MCSs indicated a higher complexity only in the oligometastatic cases. Because of an extra degree of freedom in terms of gantry positions with respect to the cranio-caudal axis along the patient, multiple coplanar lesions are treated in the same BEV of each arc more frequently than for VMAT_CDR_. This can be avoided during planning by creating independent beam sets for each lesion, such that the optimization will be carried out looking only at the dose of the separate beam set.

The calibration of the Delta^4^ was limited on the Vero system due to its 15x15 cm^2^ field size. The dosimeter relative and directional calibration were performed on another LINAC, and it can contribute to the dose discrepancies found in this work [17]. Despite the differences between the TPS and machine delivery, the DWA planned versus delivered dose distributions presented an average agreement above 98 % for all indications, DWA presenting comparable level of agreement with coplanar VMAT_CDR_. The Picket Fence test showed that the noncoplanar gantry-ring motion doesn’t affect the MLC performance (Additional file 1: Figure S1). To author’s knowledge, the ring angle variation per control point could not be integrated in the dose reconstruction of other 3D dosimetry phantoms. However, during the QA preparation process, the DWA plan can be mapped with all ring angles collapsed at 0, creating a classical coplanar VMAT which can be verified with common dosimetric phantoms available in the clinic.

For the moment, there is no module to optimize the Wave Arc trajectory directly based on dose criteria. A comparison of the current template-based approach against a geometric optimization of the DWA path is required to investigate if further improvement in therapeutic dose delivery can be provided by DWA. Nevertheless, considering the collision-free turning space of the O-ring gantry, a significant impact of inverse trajectory optimization is not expected.

The LINAC’s montage on a gimbals mechanism allows real-time tumor tracking delivery (RTTT), clinically implemented for conformal beams [11] and IMRT [12]. RTTT during DWA treatment would be a feasible extension addressing the motion management issue for abdominal tumors, since the movement of the gimbaled X-ray head and the O-ring gantry are independent.

## Conclusion

DWA combines direct machine parameter optimization with noncoplanar geometry, allowing additional flexibility in dose shaping. This study describes a preparation towards the first noncoplanar DWA treatment in clinical circumstances on the Vero system using a clinical RayStation TPS. The Vero DWA solution proven to be a fully functional treatment technique, and presented delivery times comparable with coplanar VMAT_CDR_. The initial dosimetric assessment with a 2D array provided acceptable results, however further investigations into the noncoplanar specificity of the DWA will be performed prior to clinical implementation.

## Additional file

Additional file 1: Figure S1. MLC performance. (DOCX 194 kb)

## Abbreviations

BEV: beam's eye view
DMPO: direct machine parameter optimization
DWA: Dynamic Wave Arc
LAPC: locally advanced pancreatic cancer
MCS: modulation complexity score
MLC: multi-leaf collimator
MP: manipulation points
NSCLC: non-small cell lung cancer
OAR: organs-at-risk
RTTT: real-time tumor tracking delivery
TPS: treatment planning system
VMAT: volumetric modulated arc therapy
