# Supplementary material for: Initial characterization, dosimetric benchmark and performance validation of Dynamic Wave Arc
Source: Radiat Oncol. 2016 Apr 29;11:63. doi: 10.1186/s13014-016-0633-7 (PMC4850693; doi:10.1186/s13014-016-0633-7)
Supplement: Additional file 1: Figure S1. — MLC performance. (DOCX 194 kb) [file 13014_2016_633_MOESM1_ESM.docx]

**Supplementary file**

**S1: MLC performance**

A standard picket fence test was performed to evaluate if the leaf accuracy is affected by the complex wavy motion. The test was delivered on a Gafchromic film fixed between two solid water plates of 1 cm height and attached directly to the MLC/machine cover during: a) static Gantry/Ring=0°/0° b) a 360° dynamic arc irradiation and c) a 340° dynamic wave arc irradiation. The test consisted of five 4mm wide slit-fields with 2cm interline distance. In-house software analyzed the acquired data and, for each slit, the gap position standard deviation (SD), calculated as the deviation from the mean value and the average gap width were determined.

Tolerance levels below 0.20 mm were established for the MLC positional error. Mean gap position SDs of 0.20 mm, 0.11 mm and 0.10 mm were obtained for the static, dynamic arc, and wave arc delivery respectively, together with an average gap width of respectively 3.98±0.16 mm, 4.08±0.18 mm and 4.06±0.18 mm. The Picket Fence test showed that the non-coplanar gantry-ring motion doesn’t affect the MLC performance, the lowest gap position SD being registered for DWA delivery (Figure 1), in contrast with the same configuration delivered in static mode or dynamic arc.


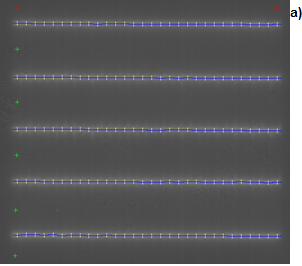

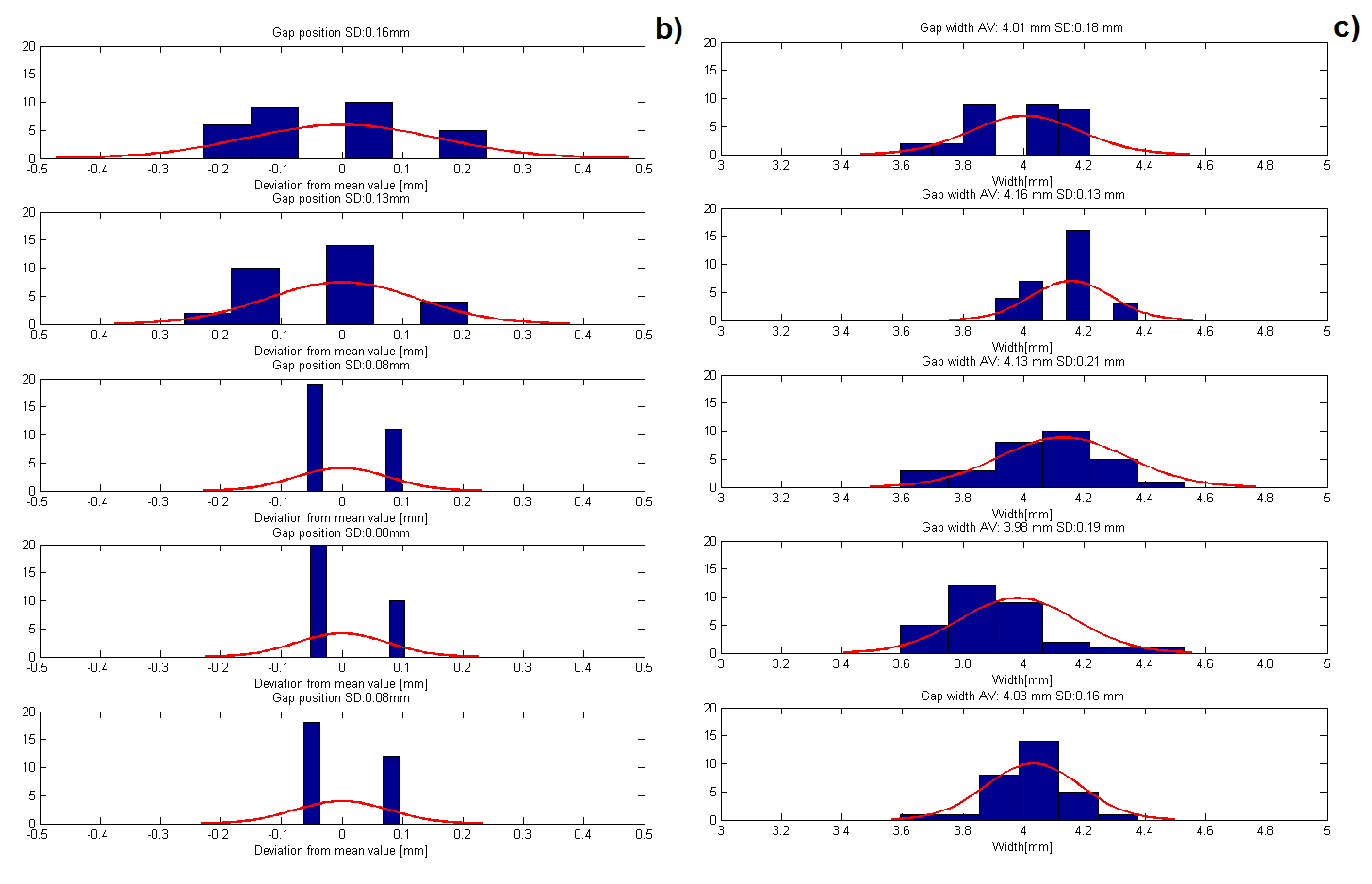


Figure 1. Picket fence delivered during DWA analysis. a) Leaf positional detection b) mean gap position SDs and c) average gap width
